# Early Phase I Study of a ^99m^Tc-Labeled Anti–Programmed Death Ligand-1 (PD-L1) Single-Domain Antibody in SPECT/CT Assessment of PD-L1 Expression in Non–Small Cell Lung Cancer

**DOI:** 10.2967/jnumed.118.224170

**Published:** 2019-09

**Authors:** Yan Xing, Gitasha Chand, Changchun Liu, Gary J.R. Cook, Jim O’Doherty, Lingzhou Zhao, Nicholas C.L. Wong, Levente K. Meszaros, Hong Hoi Ting, Jinhua Zhao

**Affiliations:** 1 Department of Nuclear Medicine, Shanghai General Hospital, Shanghai Jiaotong University School of Medicine, Shanghai, People’s Republic of China; 2 Nanomab Technology Limited, Shanghai, People’s Republic of China; 3 Department of Cancer Imaging, School of Biomedical Engineering and Imaging Sciences, King’s College London, London, United Kingdom; 4 Department of Molecular Imaging, Sidra Medicine, Doha, Qatar; and; 5 Weill Cornell Medical College, Education City, Doha, Qatar

**Keywords:** PD-L1, non–small cell lung cancer, SPECT/CT, early phase I, single domain antibody (sdAb)

## Abstract

Immunotherapy with checkpoint inhibitor programmed cell death 1 (PD-1)/programmed death ligand-1 (PD-L1) antibodies demonstrates improvements in treatment of advanced non–small cell lung cancer. Treatment stratification depends on immunohistochemical PD-L1 measurement of biopsy material, an invasive method that does not account for spatiotemporal heterogeneity. Using a single-domain antibody, NM-01, against PD-L1, radiolabeled site-specifically with ^99m^Tc for SPECT imaging, we aimed to assess the safety, radiation dosimetry, and imaging characteristics of this radiopharmaceutical and correlate tumor uptake with PD-L1 immunohistochemistry results. **Methods:** Sixteen patients (mean age, 61.7 y; 11 men) with non–small cell lung cancer were recruited. Primary tumor PD-L1 expression was measured by immunohistochemistry. NM-01 was radiolabeled with [^99m^Tc(OH_2_)_3_(CO)_3_]^+^ complex binding to its C-terminal hexahistidine tag. Administered activity was 3.8–10.4 MBq/kg, corresponding to 100 μg or 400 μg of NM-01. Whole-body planar and thoracic SPECT/CT scans were obtained at 1 and 2 h after injection in all patients, and 5 patients underwent additional imaging at 10 min, 3 h, and 24 h for radiation dosimetry calculations. All patients were monitored for adverse events. **Results:** No drug-related adverse events occurred in this study. The mean effective dose was 8.84 × 10^−3^ ± 9.33 × 10^−4^ mSv/MBq (3.59 ± 0.74 mSv per patient). Tracer uptake was observed in the kidneys, spleen, liver, and bone marrow. SPECT primary tumor–to–blood-pool ratios (T:BP) varied from 1.24 to 2.3 (mean, 1.79) at 1 h and 1.24 to 3.53 (mean, 2.22) at 2 h (*P* = 0.005). Two-hour primary T:BP ratios correlated with PD-L1 immunohistochemistry results (*r* = 0.68, *P* = 0.014). Two-hour T:BP was lower in tumors with ≤1% PD-L1 expression (1.89 vs. 2.49, *P* = 0.048). Nodal and bone metastases showed tracer uptake. Heterogeneity (>20%) between primary tumor and nodal T:BP was present in 4 of 13 patients. **Conclusion:** This first-in-human study demonstrates that ^99m^Tc-labeled anti–PD-L1-single-domain antibody SPECT/CT imaging is safe and associated with acceptable dosimetry. Tumor uptake is readily visible against background tissues, particularly at 2 h when the T:BP ratio correlates with PD-L1 immunohistochemistry results.

Non–small cell lung cancer (NSCLC) is the leading cause of cancer mortality, with nearly 1.6 million cancer-related deaths each year worldwide (1). Between 70% and 80% of patients present with unresectable advanced NSCLC at diagnosis, and despite the development of molecule-targeted therapies the prognosis of patients with advanced disease remains poor, with an overall 5-y survival rate of 15% (2,3). The recent breakthrough of immunotherapies with checkpoint inhibitor programmed cell death 1 (PD-1)/programmed death ligand-1 (PD-L1) antibodies has demonstrated improvements in efficacy and tolerability over platinum-based chemotherapy in this subset of patients (4–11). Six different PD-1/PD-L1 inhibitors have now been approved by the U.S. Food and Drug Administration for advanced NSCLC therapy.

PD-1 receptor, expressed on the surface of activated T cells, is a negative costimulatory receptor, and its ligands, PD-L1 or PD-L2, are normally expressed on the surface of dendritic cells or macrophages (12,13). Certain malignancies have developed the ability to coopt these immune checkpoints and escape the T-cell–induced antitumor activity by overexpressing PD-L1 (14,15).

Discrepancies in treatment response have highlighted potential deficiencies in the current methods to evaluate PD-L1 expression in a clinical setting (16). Immunohistochemical assessment of tumor samples obtained by core needle biopsy is often unable to capture the heterogeneity and dynamic nature of PD-L1 expression within the tumor and its microenvironment (17). In addition, core needle biopsy is not always practical (e.g., in patients with widespread metastatic or skeletal involvement) or may be of too high risk to be performed (e.g., certain mediastinal metastases). With the advent of immunotherapies, there is a need to develop a noninvasive imaging strategy to evaluate PD-L1 expression for optimal treatment stratification and monitoring.

Single-domain antibodies (sdAbs) are the smallest naturally derived antigen-binding fragments from camelid antibodies and demonstrate great potential for molecular imaging in both preclinical and clinical models (18–21). Because of their low molecular weight (∼15 kDa), sdAbs rapidly clear from the circulation via the kidneys while retaining high target-binding potential. We developed a library of hexahistidine tagged anti–PD-L1 sdAbs that specifically bind to human PD-L1 without blocking PD-1/PD-L1 interaction. Because they are designed to bind to a different domain of PD-L1, anti–PD-L1 immunotherapeutics will be unlikely to interfere with the binding of anti–PD-L1 sdAbs to PD-L1, thereby potentially allowing close monitoring of anti–PD-L1 immunotherapy by imaging. Recent studies demonstrated the potential of sdAbs for nuclear medicine imaging (22).

Our hypothesis was that SPECT imaging of NSCLC with a ^99m^Tc-labeled sdAb (^99m^Tc-NM-01) that specifically binds to human PD-L1 is feasible, is safe, and correlates with PD-L1 immunohistochemistry results. In this first-in-human study, we aimed to assess safety, radiation dosimetry, and imaging characteristics of ^99m^Tc-NM-01 and sought to compare imaging and PD-L1 immunohistochemistry results in patients with advanced NSCLC.

## MATERIALS AND METHODS

This was an open-label, nonrandomized early phase I diagnostic study in patients with untreated NSCLC (*n* = 16) between March and November 2018 (ClinicalTrials.gov identifier no. NCT02978196) and obtained approval from Shanghai General Hospital Ethics Committee (approval number: 2016KY220). All patients enrolled into this study gave written informed consent to participate.

Briefly, to radiolabel NM-01 the [^99m^Tc(OH_2_)_3_(CO)_3_]^+^ complex (pH 7.0–7.5) was added to a sealed vial containing 200 μg of NM-01 in 100 μL of phosphate-buffered saline (pH 7.4), and the mixture was incubated at 37°C for 1 h (23–25). Contents were diluted in physiologic saline; in patient group 2 (see below), to adjust the injected dose to 400 μg per patient, additional NM-01 was added in this step.

Patients aged between 18 and 75 y with histopathologically confirmed NSCLC and an Eastern Cooperative Oncology Group Performance Score of 1 or less were eligible to participate in this study (Supplemental Fig. 1; supplemental materials are available at http://jnm.snmjournals.org). PD-L1 expression was determined in primary tumor tissue obtained by core needle biopsy.

### Immunohistochemical Methodology

Primary tumor samples, 1–2 mm in diameter, were obtained by core needle biopsy for immunohistochemical evaluation of PD-L1 expression. Tissue slice controls included placental tissue as negative control and tonsil tissue as positive control, as well as confirmed positive and negative NSCLC tumor tissues. A control cell slide mounted with a PD-L1–positive and –negative cell pellet (DAKO North America, USA) was also processed in parallel. After collection, formalin-fixed paraffin-embedded blocks were prepared according to instructions set in the PD-L1 IHC 22C3 pharmDx immunohistochemical assay kit manual (DAKO North America, USA). Briefly, each specimen was fixed in 10% neutral buffered formalin. After being rinsed, samples were dehydrated by sequential immersion in ascending concentrations of ethanol in water for 2 h at each concentration, starting at 80% and reaching 100% in 5% increments. Dehydrated samples were cleared in xylene and subsequently infiltrated with melted paraffin at 55°C.

Formalin-fixed paraffin-embedded blocks were cut into 4-µm sections in an RM2235 Rotary Microtome (Leica Biosystems). Sections were mounted onto DAKO Flex IHC microscope slides (DAKO North America, USA), stored in the dark at 2°C–8°C and used within 30 d of preparation.

Further processing and immunostaining of primary tumor samples and controls were performed in a DAKO Autostainer Link 48 SK006 immunohistochemistry stainer (DAKO North America, USA) using the pre-programmed PD-L1 IHC 22C3 pharmDx staining protocol and reagents, including the mouse anti–PD-L1 (clone 22C3), provided in the assay kit. Briefly, an automated 3-in-1 process of deparaffinisation, sample rehydration, and target retrieval was followed by an automated staining procedure with mouse anti–PD-L1 (clone 22C3) antibody, followed by hematoxylin (Baso Diagnostic Inc.) counterstaining. Microscopic and histopathologic evaluations of PD-L1 immunostaining were performed on a DAKO Autostainer Link 48 (DAKO North America, USA) and results validated by a pathologist. Representative images were recorded using a Leica DM6000B microscope (Leica Biosystems) and Leica DFC 550 digital camera (Leica Biosystems).

### SPECT/CT Scan Protocol

Patients were administered ^99m^Tc-NM-01 in an intravenous bolus (3.8–10.4 MBq/kg), corresponding to 100 µg (group 1, *n* = 13; 1.7 ± 0.3 µg/kg; range, 1.2–2.1 µg/kg) or 400 µg (group 2, *n* = 3; 5.9 ± 0.3 µg/kg; range, 5.6–6.1 µg/kg) of NM-01. Patients were asked to drink 300–500 mL of water after injection then void their bladder before imaging on a GE Discovery NM07 SPECT/CT scanner (GE Healthcare). Planar scans were acquired with the patient supine at 1 and 2 h after injection at 10 cm/slice/min. A CT scan was acquired at 60 min followed by local tomographic SPECT imaging focused around primary and suspected secondary as follows. All scans were acquired using low-energy high-resolution collimators in a 20% energy window centered around 140 keV, in a 256 × 1,024 matrix for planar images and 64 × 64 matrix for tomographic images. A 10% energy window centered around 120 keV was also collected during tomographic acquisitions for attenuation and scatter correction. SPECT images were acquired over 360° in 60 frames per full rotation with 20-s acquisition per frame.

All scans were assessed by a single observer with more than 25 y of nuclear medicine and radiology experience. Maximum counts were recorded from regions of interest drawn manually on SPECT scans, using the CT for guidance, around the primary tumor; any lymph node metastases within the thoracic field of view; a 3-cm normal-lung ROI in the right upper lobe (or left upper lobe if pathology existed in the right upper lobe) for tumor-to-lung ratios (T:L); and a ROI, within the aortic arch avoiding the walls of the aorta, for calculation of tumor–to–blood-pool ratios (T:BP).

### Radiation Dosimetry Calculations

Five patients underwent extra whole-body planar imaging at additional time points (i.e., 10 min, 1 h, 2 h, 3 h, and 24 h after injection) to obtain radiation dosimetry data. A calibration source of 37 MBq at injection time was placed above the head of each patient to provide quantitative calibration of counts to activity. Visible organs were drawn on the initial anterior and posterior whole-body images for each patient at 10 min using OsiriX software (Pixmeo) by an experienced operator and transferred to all remaining time points. The remainder of body was assigned activity not accounted for by organ delineation or excretion. All organs for each patient were assumed to be of the same volume over the imaging time frame. Counts from the anterior and posterior images were geometrically averaged. Time–activity curves were converted to percentage injected dose and used as an input to the OLINDA/EXM dose calculation software (version 1.1) (26). Organ normalized cumulated activities were automatically calculated using the net injected activity and organ volume of the male standard 70-kg Cristy–Eckerman adult anthropomorphic phantom used by OLINDA/EXM. Time–activity curves were fitted to biexponential functions where possible, assuming radionuclide decay (i.e. no further excretion) on reaching the last imaging point. Red bone marrow dosimetry was calculated from outlining of the lumbar vertebrae and using a well-known model whereby the lumbar vertebrae contain 12.3% of red bone marrow in adults (27). The normalized cumulated activity of the urinary bladder contents was calculated using a dynamic urinary bladder model in the OLINDA/EXM using a 3.5-h voiding interval as recommended by the International Commission on Radiological Protection.

### Statistics

Differences and correlations between parameters were tested using the Wilcoxon signed-rank test, Mann–Whitney *U* test, and Spearman correlation using IBM SPSS Statistics (version 24) software. A *P* value of less than 0.05 was taken for statistical significance.

## RESULTS

Average radiochemical purity of radiolabeled NM-01 was 96.9% ± 0.9% (95.4%–98.5%); purification after radiolabeling was not necessary (Supplemental Fig. 2). Endotoxin levels remained below 0.4 EU/mL.

### Patient Characteristics

Sixteen patients (11 men; mean age, 61.7 y) with histopathologically proven NSCLC (9 squamous cell carcinoma, 7 adenocarcinoma) were included. Clinical stage and other characteristics are summarized in Table 1. Uptake in lymph node metastases was observed in 12 patients. PD-L1 expression determined by immunohistochemistry ranged from 0% to 85% (Supplemental Fig. 3). The mean administered activity of ^99m^Tc-NM-01 was 372 ± 62 MBq (range, 255–485 MBq) in group 1 and 659 ± 25 MBq (range, 635–685 MBq) in group 2 (Table 1).

**TABLE 1:** Patient Characteristics

Dose group	Patient no.	Age (y)	Sex	Tumor type	Tumor size (CT axial dimensions)	Clinical staging	PD-L1 expression (%)	ECOG score
Group 1 (3.8–8.4 MBq/kg, 1.2–2.1 μg/kg)	1	49	Male	Adenocarcinoma	37 × 27 mm	cT4N3M1 IV	NA	1
	2	75	Male	Squamous cell carcinoma	44 × 48 mm	T3N3M1 IV	20	1
	3	75	Male	Squamous cell carcinoma	55 × 46 mm	T2bN3M0 IIIB	0	1
Group 2 (9.1–10.4 MBq/kg, 5.6–6.1 μg/kg)	4	65	Male	Adenocarcinoma	48 × 42 mm	T2bN3M1 IV	0	0
	5	57	Male	Squamous cell carcinoma	32 × 35 mm	cT2N2M0 IIIA	55	0
	6	65	Male	Squamous cell carcinoma	30 × 58 mm	T2aN2M0 IIIA	3	0
Group 1 (3.8–8.4 MBq/kg, 1.2–2.1 μg/kg)	7	75	Female	Adenocarcinoma	38 × 28 mm	T2aN0M0	NA	0
	8	52	Female	Squamous cell carcinoma	33 × 23 mm	T2aN0M0 1B	0	0
	9	36	Female	Adenocarcinoma	45 × 35 mm	T2aN2M1 IV	1	1
	10	46	Female	Adenocarcinoma	42 × 35 mm	T3N1M0 IIIA	50	0
	11	51	Male	Squamous cell Carcinoma	47 × 35 mm	T2aN3M0 IIIB	2	0
	12	72	Male	Adenocarcinoma	46 × 53 mm	T2bN3M1 IV	NA	1
	13	55	Male	Squamous cell carcinoma	71 × 78 mm	T4N0M1c IV	85	0
	14	69	Male	Squamous cell carcinoma	20 × 28 mm	T3N1M0 IIIA	10	0
	15	71	Female	Squamous cell carcinoma	78 × 95 mm	T4N1M1a IV	NA	1
	16	60	Male	Adenocarcinoma	93 × 75 mm	T4N3M1a IV	2	0

ECOG = Eastern Cooperative Oncology Group Performance Score; NA = not available.

### Safety Assessment

^99m^Tc-NM-01 was administered in 16 patients; no drug-related adverse reactions were reported during the 7-d follow-up period. Vital signs were measured during the screening period, before injection and then every 60 min up to 5 h after injection and first follow up period (48 h after SPECT/CT scan). Clinical laboratory testing, including standard hematologic and comprehensive metabolic panels (i.e., total red blood cell count, total white blood cell count, neutrophil count, lymphocyte count, platelet count, plasma creatinine, plasma blood urea nitrogen, plasma ionized calcium, sodium, potassium, lactate dehydrogenase, alanine aminotransferase, aspartate aminotransferase, alkaline phosphatase, total serum bilirubin, and serum albumin), was performed during the screening period and 48-h follow-up period. In an additional step to ensure the safety of patients, a phone interview was conducted 7 d after the SPECT/CT scan to follow-up on patients, and a questionnaire was filled out by the investigator as presented in the case report form. Symptoms related to advanced-stage lung cancer, that is, frequent coughing, dyspnea, and fatigue, reported by some patients were not attributed to the radiopharmaceutical by the supervising physician. Blood and urine clinical laboratory tests performed in the follow-up period were in the reference range of National Cancer Institute–Common Toxicology Criteria for Adverse Events (version 4.0_ standards. There were no significant changes recorded in clinical parameters such as heart rate, respiratory rate, body temperature and blood pressure during the follow-up. After participating in this study, patients proceeded with their treatment regimen.

### Biodistribution

**FIGURE 1.**
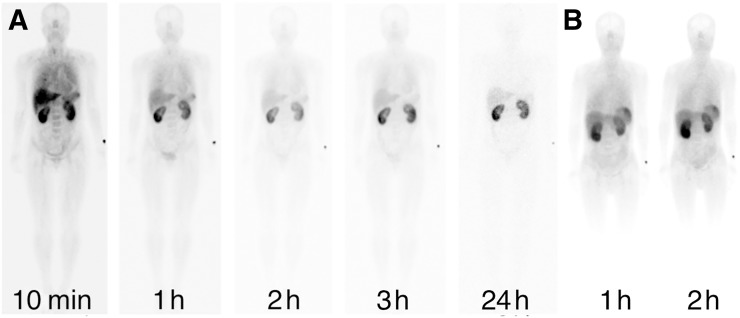
Anterior whole-body images of patient at 10 min and 1, 2, 3, and 24 h after injection of 100 μg (group 1) (A) and 1- and 2-h images of a patient administered 400 μg of NM-01 (group 2) (B). No significant difference in biodistribution was seen between the 2 groups.

Tracer biodistribution in normal organs reflected physiologic expression of PD-L1, with uptake observed in the lungs, liver, spleen, and bone marrow (Fig. 1) (28,29). Activity in these organs reduced with time and, in particular, lung and blood-pool activity reduced between 1 and 2 h in all patients, allowing increased tumor-to-background conspicuity at 2 h. Early diffuse lung activity that subsequently reduces may be as a result of a combination of blood pool and marginating immune cell activity (30). Renal activity persisting over 24 h reflects excretion and possible renal tubular retention (Fig. 1).

No noticeable differences in image quality and no statistically significant differences in tumor-to-background ratio were observed between group 1 and group 2 (Fig. 1).

### Radiation Dosimetry

**FIGURE 2.**
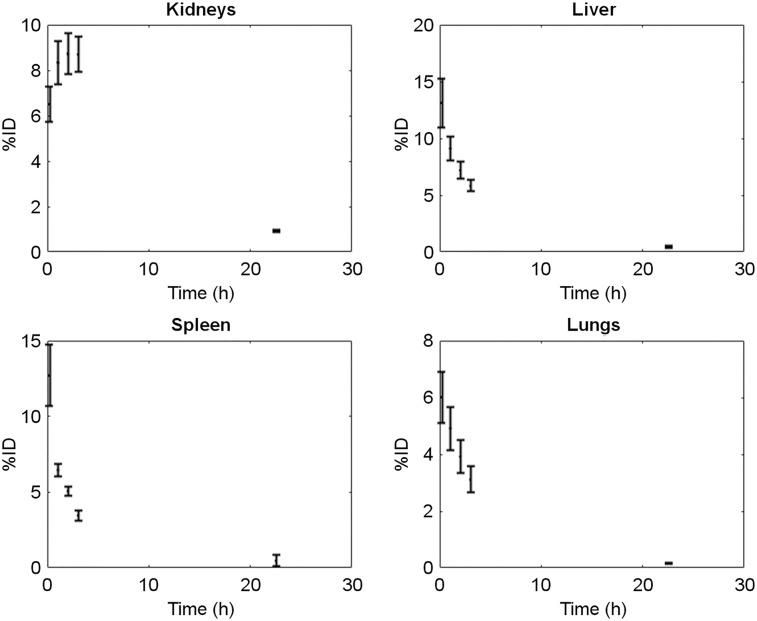
Time–activity curves for organs with highest radiotracer uptake.

Table 2 summarizes individual organ doses and effective doses for patients included in the dosimetry study (*n* = 5). All patients had normal liver and kidney functions. The kidneys showed the highest organ dose (0.036 ± 0.018 mSv/MBq), followed by the bladder (0.026 ± 0.011 mSv/MBq), spleen (0.022 ± 0.011 mSv/MBq), and liver (0.011 ± 0.0025 mSv/MBq) and in line with the uptake and excretory pathway of the imaging agent. Time–activity curves for organs with highest radiotracer uptake are shown in Figure 2.

**TABLE 2:** Organ Radiation Doses

Organ/tissue	Mean (mSv/MBq)	SD (mSv/MBq)
Adrenals	5.54E−03	1.80E−03
Brain	1.41E−03	4.15E−04
Breasts	1.66E−03	5.86E−04
Gallbladder	4.89E−03	1.69E−03
Lower large intestine wall	4.00E−03	9.79E−04
Small intestine	3.88E−03	1.13E−03
Stomach	6.04E−03	1.71E−03
Upper large intestine wall	3.72E−03	1.15E−03
Heart wall	3.17E−03	1.04E−03
Kidneys	3.60E−02	1.76E−02
Liver	1.10E−02	2.51E−03
Lungs	4.53E−03	1.23E−03
Muscle	2.62E−03	8.35E−04
Ovaries	4.11E−03	1.01E−03
Pancreas	5.72E−03	2.01E−03
Red marrow	6.73E−03	3.25E−03
Osteogenic cells	8.08E−03	1.74E−03
Skin	1.49E−03	5.46E−04
Spleen	2.21E−02	1.14E−02
Testes	2.44E−03	8.40E−04
Thymus	2.32E−03	7.89E−04
Thyroid	4.00E−03	2.25E−02
Urinary bladder wall	2.58E−02	1.05E−02
Total body	3.20E−03	9.13E−04
Effective dose	8.84E−03	9.33E−04

Radiation-absorbed doses calculated by OLINDA/EXM software and overall effective dose (mSv/MBq) (*n* = 5).

### Tumor Activity and Relationship with PD-L1 Immunohistochemistry

**FIGURE 3.**
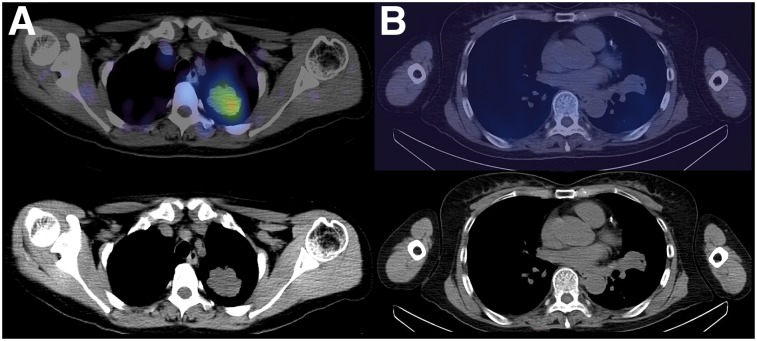
(A) Left upper lobe tumor T:BP = 3.12 (PD-L1 expression 50%). (B) Left upper lobe tumor T:BP = 2.26 (PD-L1 expression 0%).

**FIGURE 4.**
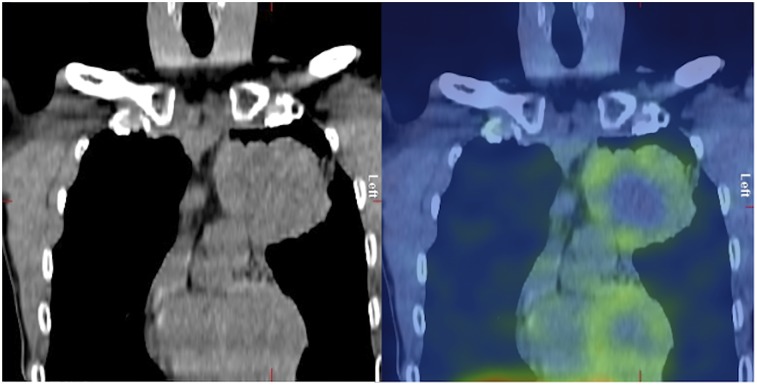
Left upper lobe tumor showing heterogeneity of PD-L1 expression. Central photopoenia is in keeping with necrosis, but there is heterogeneity of uptake in solid peripheral component of tumor (T:BP = 2.46, PD-L1 expression 85%).

The primary tumor T:L ratio was greater at 2 h than 1 h (mean, 2.69 vs. 2.22, *P* = 0.034), allowing reasonably good tumor conspicuity against normal background lung activity in patients with high PD-L1 expression (Fig. 3; Table 3). The primary tumor T:BP ratio was also greater at 2 h (mean, 2.22 vs. 1.79, *P* = 0.005). Intratumoral heterogeneity of tracer uptake was noted in some primary tumors (Fig. 4). Patients with a PD-L1 expression of 1% or less, a level often used to stratify patients, showed significantly lower T:BP ratios (mean, 1.89 vs. 2.49, *P* = 0.048). When receiver operating characteristic analysis is used, this gives an optimal threshold for the 2-h T:BP of 2.32 (area under the receiver operating characteristic, 0.88).

**TABLE 3:** Imaging Characteristics

Patient no.	sdAb dose group	Injected activity (MBq)	SPECT T:L ratio 1 h	SPECT T:L ratio 2 h	SPECT T:BP ratio 1 h	SPECT T:BP ratio 2 h	SPECT highest lymph node T:BP ratio 1 h	SPECT highest lymph node T:BP ratio 2 h
1	1	339	1.92	2.17	1.31	1.24	1.84	1.64
2	1	374	2.82	2.99	2.03	3.09	1.99	3.40
3	1	375	2.16	2.80	1.25	1.65	1.31	1.73
4	2	656	1.19	1.44	1.24	1.66	1.43	1.73
5	2	685	0.93	1.10	2.23	2.65	1.65	1.95
6	2	635	2.71	1.88	1.75	1.79	2.22	3.24
7	1	255	1.80	2.06	1.31	1.76	NP	NP
8	1	398	2.48	2.41	1.83	2.26	NP	NP
9	1	486	1.42	2.07	1.95	2.00	2.1	1.9
10	1	381	3.15	5.63	2.13	3.12	1.47	1.75
11	1	317	1.92	1.74	1.73	2.37	1.39	2.26
12	1	448	4.17	6.50	2.20	3.53	3.05	3.13
13	1	400	2.4	3.09	1.61	2.46	NP	NP
14	1	409	1.49	1.54	1.68	1.98	1.75	1.34
15	1	289	1.69	1.47	2.16	1.55	1.9	1.77
16	1	363	3.35	4.15	2.3	2.47	2.23	2.09
Mean			2.22	2.69	1.79	2.22	1.83	2.02
Median			2.04	2.12*	1.79	2.13^†^	1.84	1.77^‡^

* *P* = 0.034 between 1 and 2 h.

† *P* = 0.005 between 1 and 2 h.

‡ *P* = not significant.

NP = not present.

**FIGURE 5.**
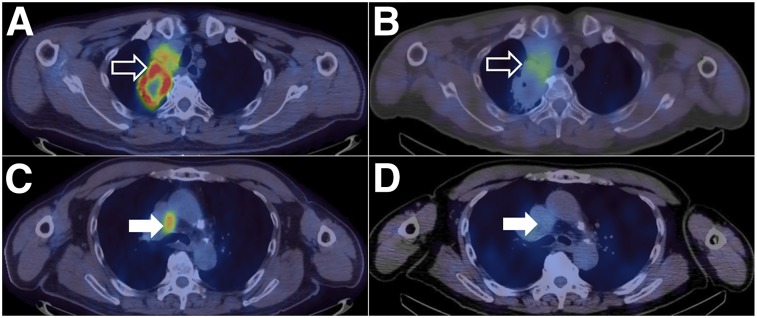
Right upper lobe tumor (open arrows) shows areas of high ^18^F-FDG uptake (SUV_max_ = 16.1) on PET/CT (A) and ^99m^Tc-SPECT/CT (T:BP = 3.53) (B). Mediastinal lymph nodes (closed arrows) show high ^18^F-FDG uptake (SUV_max_ = 6.3) (C) but low ^99m^Tc-NM-01 activity (T:BP = 1.13) (D), demonstrating heterogeneity of PD-L1 expression between primary tumor and nodal sites of disease within same patient.

**FIGURE 6.**
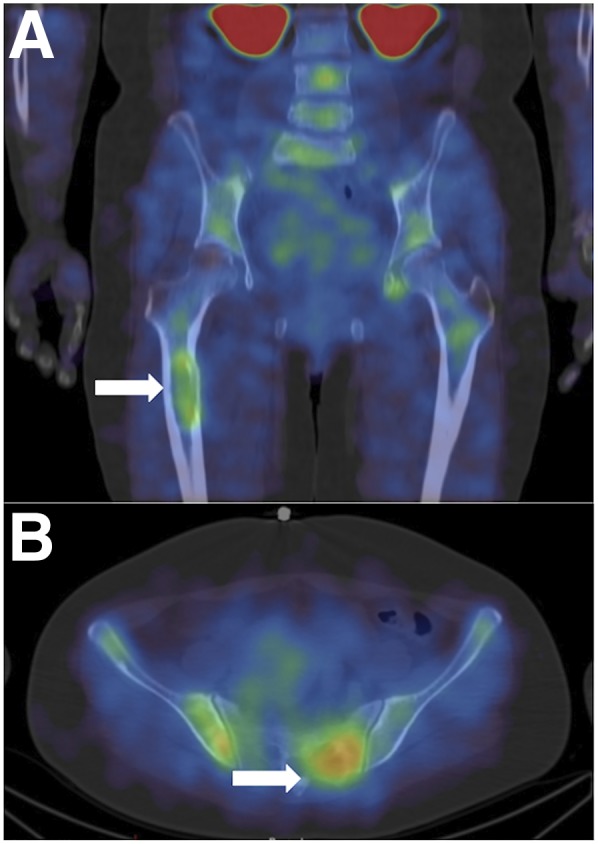
Coronal (A) and axial (B) ^99m^Tc-NM-01 SPECT/CT images of a patient with skeletal metastases (arrows) demonstrating PD-L1 expression.

Thoracic lymph node metastases T:BP ratios were also greater at 2 h, although this difference did not reach statistical significance (mean, 2.02 vs. 1.83; *P* = 0.12). At 2 h, primary tumor and maximum lymph node metastasis T:BP ratios (*n* = 13) showed a correlation (*r* = 0.69, *P* = 0.013). Intrapatient heterogeneity of T:BP ratios was noted between the primary tumor and lymph node metastases in several patients with a mean difference of 19% (±17%), with 4 of the 13 patients with lymph node metastases showing a greater than 20% difference (Fig. 5). If a 2-h T:BP of 2.32 is used as a threshold for positivity, then only 3 of the 13 patients with nodal disease would have been PD-L1–positive on SPECT. One patient with known bone metastases showed ^99m^Tc-NM-01 uptake (Fig. 6). Primary tumor T:BP ratios at 2 h correlated with PD-L1 immunohistochemistry results (*r* = 0.68, *P* = 0.014). Statistically significant correlations were not found for T:L ratios or 1-h T:BP ratios.

## DISCUSSION

This early phase 1 study demonstrates that ^99m^Tc-NM-01 SPECT/CT imaging is a safe procedure, with no adverse events due to the radiopharmaceutical recorded in this cohort of patients. We achieved reproducibly high radiochemical purity in labeling NM-01. Radiation dosimetry is acceptable at 8.84 × 10^−3^ ± 9.33 × 10^−4^ mSv/MBq (3.59 ± 0.74 mSv per patient) and similar to other SPECT agents in clinical use. The kidneys received the highest organ dose (0.036 ± 0.018 mSv/MBq), being the major route of excretion, and although it remained tolerable, urinary tract dose could be further minimized by good hydration or coinjection of amino acids or peptides (31).

Patients were administered either 100 or 400 μg of protein to explore specific binding in the liver and spleen, but no significant impact was observed in image quality or organ biodistribution. No statistically significant differences were noted in tumor-to-background ratios between the groups, therefore, subsequent studies were performed with 100 μg.

Biodistribution was as expected with early activity in the liver, spleen, and kidneys and some activity in the lungs and bone marrow (28). Rapid renal excretion allowed reduction of nonspecific background blood-pool and organ activity and enabled imaging at relatively early time points. In particular, rapid reduction in normal lung and blood-pool activity allowed sufficient conspicuity of primary NSCLC and metastases on SPECT imaging. Imaging characteristics were more favorable at 2 h after injection than at 1 h because of reduction in lung and blood-pool activity over this time.

Measurement of PD-L1 expression by immunohistochemistry is currently the method with which patients are stratified for anti–PD-1/PD-L1 immunotherapy. Because of the heterogeneity of PD-L1 expression in lung cancers, biopsy material from 1 region of a tumor may not allow global or other regional assessment of PD-L1 expression (32). The underestimation of PD-L1 expression by sampling error in heterogeneous tumors may be one of the reasons that apparent PD-L1–negative tumors respond to PD-1/PD-L1 checkpoint inhibitor therapy (33–35). Immunohistochemical evaluation is also limited by practicality and safety of multiple biopsies in patients with metastatic disease. Relying on immunohistochemistry results from tissue biopsy also limits the practicality of observing the evolution of PD-L1 expression in primary and metastatic tumors during therapy. For these reasons, a noninvasive imaging method that is safe and associated with low radiation dose, that can accurately report locoregional PD-L1 expression in primary tumors and metastases at serial time points, would be a valuable tool for guiding clinical management of patients with PD-L1–positive malignancies.

The use of ^99m^Tc, with a half-life of 6 h and a straightforward site-specific labeling technique for NM-01, would allow distribution from centralized radiopharmacies as well as on-site labeling at any standard hospital radiopharmacy, enabling widespread use of this methodology. There would also be future potential for labeling with PET radionuclides to help improve image spatial resolution, contrast, and quantification (32).

Our initial results show promise that the simple measurement of T:BP ratios at 2 h correlates with PD-L1 expression and if confirmed in subsequent studies could potentially replace or complement immunohistochemistry in some circumstances. We noted heterogeneity of activity within primary tumors in some patients, and between the primary tumor and metastases within patients, suggesting that intra- and intertumoral heterogeneity of PD-L1 expression is relatively common and is another factor that could be measured noninvasively with imaging to allow more relevant information to be available for patient treatment decisions. Intratumoral PD-L1 expression heterogeneity may also explain the imperfect correlation with T:BP (*r* = 0.68).

A potential limitation of this study is the relatively small number of patients that was included, although it is similar to other phase 1 studies of radiopharmaceuticals and has sufficient power to allow statistical comparisons of optimal imaging time, radiodosimetry calculations, and correlations with immunohistochemistry results. Immunohistochemical analysis was not available in some patients, and only primary tumors were assessed, thus not allowing comparison between imaging and PD-L1 expression in nodal and distant metastases. SPECT images were acquired for up to 2 h after injection in all patients. To determine whether image characteristics (i.e., target-to-background) could be improved, further scanning beyond 2 h could be considered in subsequent studies. The relatively low spatial resolution of SPECT does not allow more than gross visual intratumoral heterogeneity observations. Although a PET tracer, in theory, might allow more accurate and detailed heterogeneity analysis similar to what has been reported with ^18^F-FDG PET/CT in NSCLC (36), recent studies with an anti–PD-L1 tracer for PET also reported on insufficient spatial resolution (32). In addition, we have examined only NSCLC in this study and cannot extrapolate these results to other malignancies that are known to express PD-1/PD-L1.

## CONCLUSION

This study demonstrates that ^99m^Tc-labeled anti-PD-L1-sdAb SPECT/CT using ^99m^Tc-NM-01 is a safe diagnostic procedure, delivering a tolerable radiation dose and presenting favorable biodistribution and image characteristics correlating with PD-L1 immunohistochemistry results in patients with NSCLC. As PD-1/PD-L1 immune checkpoint inhibitors are more frequently used in the treatment of NSCLC, a companion imaging methodology is becoming increasingly sought after for the staging and monitoring of patients. Anti–PD-L1-sdAb SPECT/CT using ^99m^Tc-NM-01 has the potential to be used for the close monitoring of changes in PD-L1 expression levels during PD-L1 immunotherapy. In addition, the heterogeneous and dynamic nature of PD-L1 expression in most malignancies could make imaging a preferred method, compared with more invasive core needle biopsies, in diagnosing and staging patients with PD-L1–positive malignancies.

## DISCLOSURE

This research is supported by Nanomab Technology Limited and Shanghai Shenkang Development Center for Clinical Skills Innovation (16CR3052A). The authors acknowledge financial support from the King’s College London/University College London Comprehensive Cancer Imaging Centres funded by Cancer Research U.K. and Engineering and Physical Sciences Research Council in association with the Medical Research Council and the Department of Health (C1519/A16463) and the Wellcome Trust EPSRC Centre for Medical Engineering at King’s College London (WT203148/Z/16/Z). No other potential conflict of interest relevant to this article was reported.

## KEY POINTS

**QUESTION:** ^99m^Tc-labeled anti–PD-L1-sdAb SPECT/CT imaging is safe in humans and can show PD-L1 expression in primary tumors and metastatic malignancies, with numerous cases exhibiting tumoral heterogeneity.
**PERTINENT FINDINGS:** This early phase 1 open-label nonrandomized diagnostic study in 16 patients showed no drug-related adverse events, demonstrating safety of the radiotracer in the cohort of patients. Tumor–to–blood-pool ratios correlated with PD-L1 immunohistochemistry results overall, though heterogeneity was seen between nodal and bone metastatic malignancies within the same patient, proving that PD-L1 expression detection by immunohistochemical method may not be an optimal method.
**IMPLICATIONS FOR PATIENT CARE:** Patients could potentially use ^99m^Tc-labeled anti–PD-L1-sdAb SPECT/CT as a fast and noninvasive tool to gain insight to PD-L1 expression of primary tumor as well as metastatic malignancies and observe for heterogeneity within the tumor and metastases; this tool could ultimately be used as a prognostic and treatment evaluation tool for checkpoint inhibitor therapy.
