# Supplementary material for: Early Phase I Study of a 99mTc-Labeled Anti–Programmed Death Ligand-1 (PD-L1) Single-Domain Antibody in SPECT/CT Assessment of PD-L1 Expression in Non–Small Cell Lung Cancer
Source: J Nucl Med. 2019 Sep;60(9):1213–20. doi: 10.2967/jnumed.118.224170 (PMC6735283; doi:10.2967/jnumed.118.224170)
Supplement: Supplementary file 1 [file jnm224170SupplementalData.pdf]

Patient recruitment, intervention allocation, follow up and analysis are described in Supplemental Figure 1.

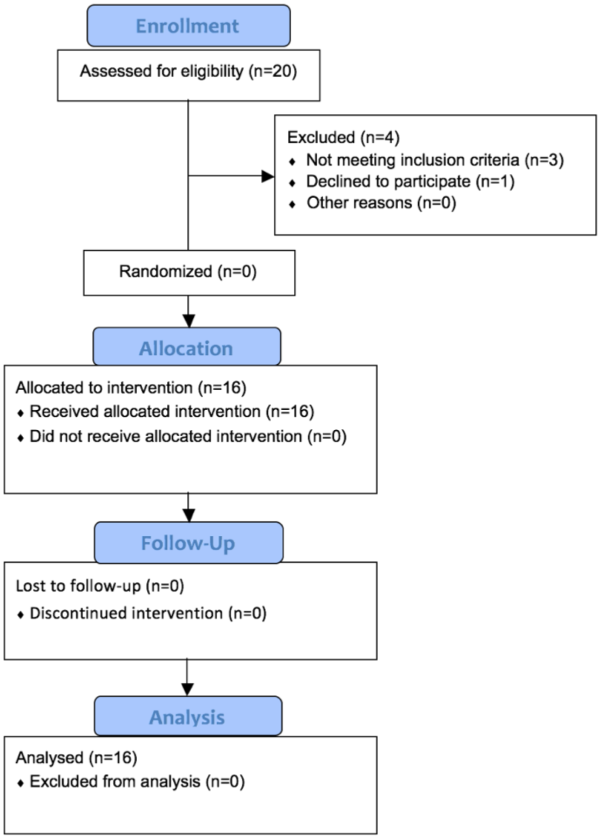

Supplemental Figure 1: CONSORT flowchart of patient enrollment, allocation, follow-up and analysis.

NM-01 production and formulation

The anti-PD-L1 sdAb NM-01 was produced to good manufacturing practice (GMP). The sequence with carboxy terminal hexahistidine tail was subcloned in expression vector pE7 and produced in

Escherichia coli BLR(DE3). The expressed sdAb was further purified from periplasmic extracts by cation exchange chromatography on SP Sepharose (GE Healthcare, USA) and buffer exchanged to phosphate buffered saline (PBS) with gel filtration on Superdex 75 resin (GE Healthcare, USA). sdAb concentration was adjusted to 2.0 mg/ml in PBS and 100 µl NM-01 aliquots, containing 200 µg NM-01 were aseptically dispensed into septum sealed sterile glass vials. Aliquots were stored at -80°C until use.

Preparation of the $[^{99m}Tc(OH_2)_3(CO)_3]^+$ complex

All ingredients used in the preparation of $[^{99m}Tc(OH_2)_3(CO)_3]^+$ complex and subsequent radiolabeling of NM-01 were sterile and prepared to GMP standards. Sodium pertechnetate in physiological saline was obtained from a $^{99}Mo/^{99m}Tc$ generator. The $[^{99m}Tc(OH_2)_3(CO)_3]^+$ complex was prepared in solution using an in situ CO source (*3*). To a septum-sealed glass vial containing 4.5 mg $Na_2[H_3BCO_2]$, 2.85 mg $Na_2B_4O_7{\cdot}10H_2O$, 7.15 mg Na_2CO_3 and 8.5 mg $Na_2C_4H_4O_6{\cdot}2H_2O$ under argon 2146-4810 MBq sodium pertechnetate were added in 1.0 ml physiological saline. The vial was incubated in a boiling waterbath for 30 minutes. Following incubation, the vial was cooled to room temperature and 180 µl 1.0 M HCl were added to bring the pH to 7.0-7.5.

QC of the $[^{99m}Tc(OH_2)_3(CO)_3]^+$ complex was performed by thin layer chromatography (TLC) in a system consisting of silica gel 60 F254 TLC plates (Merck, Germany) and 1% HCl in methanol mobile phase. In this TLC system colloidal ^{99m}Tc has a retention factor (R_f) of 0; $[^{99m}Tc(OH_2)_3(CO)_3]^+$ is represented by a broad peak with an average R_f=0.4-0.5 and $^{99m}TcO_4^-$ has an R_f=1. Only $[^{99m}Tc(OH_2)_3(CO)_3]^+$ with a radiochemical purity (RCP) >95% was used for NM-01 radiolabeling. In this step RCP was defined as ^{99m}Tc radioactivity associated with $[^{99m}Tc(OH_2)_3(CO)_3]^+$ as a percentage of total ^{99m}Tc radioactivity on the plate.

Radiolabeling NM-01 and preparation of patient doses

To a 200 µg aliquot of NM-01 in 100 µl sterile PBS 400 µl $[^{99m}Tc(OH_2)_3(CO)_3]^+$ pH 7.0-7.5, corresponding to 670-1510 MBq were added. The vial was incubated at 37°C for 1 hour. In group 1, 1.5 ml saline were added to the kit vial containing ^{99m}Tc-NM-01 after incubation, bringing the total volume to 2.0 ml. In group 2, in place of 1.5 ml saline 500 µg NM-01 in 1.25 ml saline were added to the kit vial after incubation, bringing the total volume to 1.75 ml. QC tests, including visual check and assessment of RCP, pH measurement and evaluation of endotoxin levels were performed. RCP testing was routinely performed by TLC in a system consisting of silica gel coated instant thin layer chromatography (ITLC-SG) plates (Agilent Technologies, USA) and citrate buffer pH 5.4 mobile phase. In this system ^{99m}Tc radiolabeled NM-01 has R_f=0 and unreacted $[^{99m}Tc(OH_2)_3(CO)_3]^+$ and $^{99m}TcO_4^-$ both have R_f=1. ^{99m}Tc-NM-01 with RCP>95%, pH=7.0-7.5, endotoxin levels <15 EU/ml with a colorless, clear appearance was deemed acceptable for use in this study. RCP in this step was defined as ^{99m}Tc radioactivity associated with NM-01 as a percentage of total ^{99m}Tc radioactivity on the plate. Since RCP exceeded 95% in every case purification after radiolabeling was not necessary. For patient injection 1.0 ml of the final solution, corresponding to 286 – 546 MBq/100 µg ^{99m}Tc-NM-01 (group 1) or 697 - 736 MBq/400 µg ^{99m}Tc-NM-01 was withdrawn aseptically into a 2.0 ml syringe.

High performance liquid chromatography (HPLC) characterization of ^{99m}Tc-NM-01

The radiolabeled NM-01 was characterized by size exclusion (SEC) chromatography. SEC HPLC analyses were performed in an Agilent 1200 HPLC system (Agilent Technologies, USA) consisting of a manual injector and quaternary solvent pump, coupled to a Phenomenex BioSep

SEC-s2000, 5 μm, 145A, 300x7.8 mm column fitted with a Phenomenex Securityguard GFC2000 4x3 mm guard cartridge (both Phenomenex, USA), connected to a single channel variable wavelength detector (Agilent Technologies, USA) and a BioScan Flowcount gamma detection unit (BioScan, USA). Analyses were performed in isocratic 45% acetonitrile – 0.1% trifluoroacetic acid in water at a flow rate of 1 ml/min over 30 minutes with UV detection at 280 nm. In this system the ^{99m}Tc-NM-01 is represented by a single peak at 6min 47 sec ± 2 sec (n=4), the $[^{99m}Tc(OH_2)_3(CO)_3]^+$ complex is represented by a single peak at 10min 40 sec ± 2 sec (n=3) and $^{99m}TcO_4^-$ is represented by a single peak at 21min 34 sec ± 20 sec (n=4). A representative HPLC radiochromatogram of ^{99m}Tc-NM-01 is presented in Supplemental Figure 2. Radiolabeling and quality control method development will be published elsewhere.

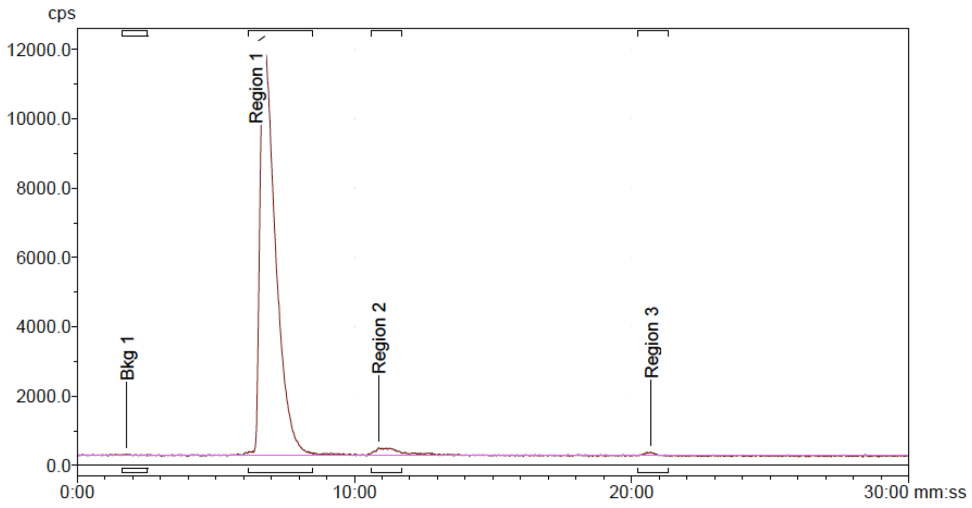

Supplemental Figure 2: HPLC radiochromatogram of ^{99m}Tc-NM-01 (Region 1: ^{99m}Tc-NM-01 (6:47, 97.7%), Region 2: [$^{99m}Tc(OH_2)_3(CO)_3]^+$ (10:47, 2.0%), Region 3: $^{99m}TcO_4^-$ (20:52, 0.3%).

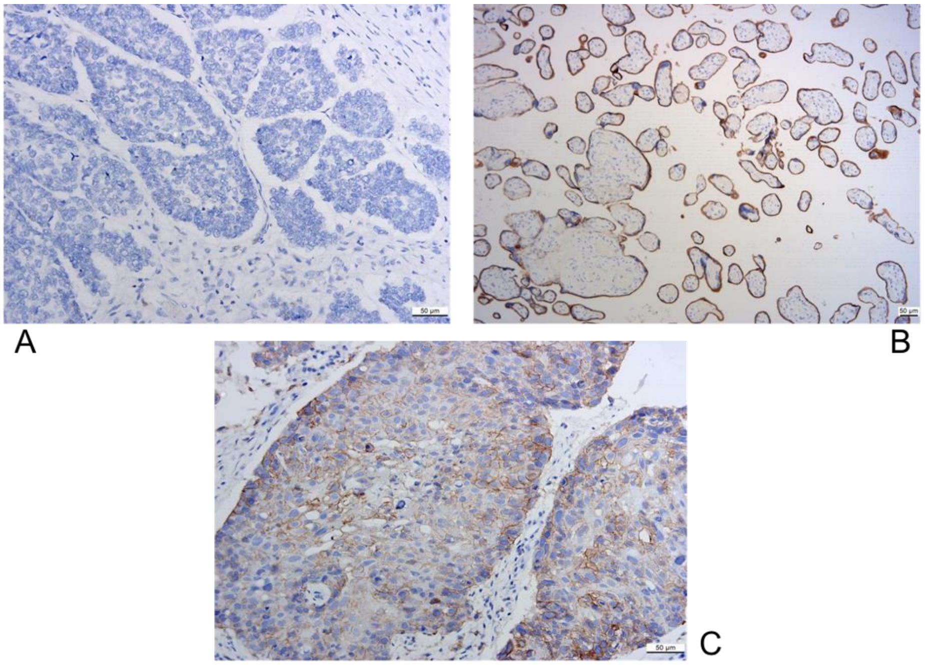

Supplemental Figure 3: PD-L1 immunohistochemical staining of (A) negative control, (B) positive control and (C) Patient 5 primary tumor tissue showing 55% expression.

Reference:

1. Alberto R, Schibli R, Egli A, Schubiger AP, Abram U, Kaden TA. A Novel Organometallic Aqua Complex of Technetium for the Labeling of Biomolecules: Synthesis of [99mTc(OH2)3(CO)3]+ from [99mTcO4]- in Aqueous Solution and Its Reaction with a Bifunctional Ligand. *Journal of the American Chemical Society.* 1998;120:7987-7988.
